# Carbon Peaking and Carbon Neutrality in the Cement-Based Materials

**DOI:** 10.3390/ma16134705

**Published:** 2023-06-29

**Authors:** Gui-Yu Zhang, Xiao-Yong Wang

**Affiliations:** 1 Department of Integrated Energy and Infra System, Kangwon National University, Chuncheon-si 24341, Republic of Korea; zhangguiyu@kangwon.ac.kr; 2 Department of Architectural Engineering, Kangwon National University, Chuncheon-si 24341, Republic of Korea

The cement industry plays a significant role in global carbon emissions, accounting for approximately 8% of global anthropogenic carbon dioxide (CO_2_) emissions [1]. In addition, 1.7 tons of raw materials are needed to produce 1 ton of clinker. Addressing the environmental impact of cement-based materials is critical as we work to combat climate change. Achieving carbon peaking and carbon neutrality in this industry is vital to sustainable development and presents opportunities for innovation and progress.

The carbon peaking of cement-based materials refers to the point at which CO_2_ emissions from the cement industry reach their highest level and gradually decline. A multifaceted approach must be taken to achieve carbon peaking in the cement industry. This includes implementing energy-saving technologies, optimizing production processes, and reducing dependence on fossil fuels. Furthermore, promoting circular economy principles and using waste materials (such as fly ash and slag) as alternative cementitious materials can further reduce the carbon footprint of cement-based materials. Carbon neutrality of cement-based materials refers to achieving a balance between the inflow of CO_2_ into the atmosphere and the removal of CO_2_. In cement-based materials, this can be achieved through various strategies. Carbon capture and storage (CCS) technology offers a promising avenue to capture and store CO_2_ emissions from cement plants. By investing in R&D and fostering collaboration between academia, industry, and government, we can accelerate the deployment of CCS technologies to achieve carbon neutrality. Additionally, investing nature-based solutions, such as reforestation and sustainable land use practices, can help offset remaining emissions and further contribute to carbon neutrality.

This Special Issue, “Carbon Peaking and Carbon Neutrality in Cement-Based Materials”, highlights the recent advances in research on cement-based and alkali-excited materials. These developments are as follows. Zia et al. [2] reviewed cement-based composites by adding waste tire steel fibers. It was found that waste tire steel fibers increased the compressive strength, splitting tensile strength, and flexural strength of cement mortar by 46%, 50.6%, and 69%, respectively. Lee et al. [3] conducted a study on manufacturing concrete using a mixture of ordinary Portland cement, early Portland cement, and granulated blast furnace slag. Their results found that the combined use of early Portland cement and granulated blast furnace slag significantly improved the concrete’s early strength and chloride ion resistance. Yang et al. [4] used various experimental methods to study the high-temperature resistance of cement-based materials added with biochar. Their research showed that as the biochar content increased, cracks in samples exposed to high temperatures decreased. Han et al. [5] investigated the properties of binary, ternary, and quaternary mixtures. Their research found that the multi-component mixture has great advantages in durability and sustainability, and has good development prospects.

Fu et al. [6] studied the properties of engineering cement-based composites. Their research shows that the amount of fly ash and high-strength polyethylene can measure the performance and cost of green lightweight engineering cement-based composites. When the amount of fly ash beads is 0.45 (fly ash beads/binder) and the amount of high-strength polyethylene is 1%, the cost is reduced by 40% and the carbon emission is reduced by 36%. Chen et al. [7] studied the frost resistance of cement–soil with lithium slag added. Their research results found that when the addition of lithium slag was 12%, the internal pore structure of cement–soil could be optimized, and the expansion of frost heave cracks caused by freeze–thaw cycles could be inhibited. Umar et al. [8] studied the mechanical properties of sustainable concrete incorporating industrial waste. Their results showed that the use of 15% coal bottom ash and 10% waste glass sludge as cementitious additives and cement substitutes has the potential to increase the strength of concrete significantly. Istuque et al. [9] evaluated the reactivity of metakaolin-based polymers using electrical impedance spectroscopy. Their study found that the electrical impedance spectroscopy technique showed excellent sensitivity in identifying changes in the polymerization process of different batches of metakaolin. David et al. [10] studied the performance of recycled slag and fly ash by adding 0.1–0.5% calcium sulfate. Their research found that calcium sulfate promoted the hydration reaction of the mixture and that a higher calcium sulfate content increased the amount of ettringite that formed, increased the strength, and shortened the setting time. Other researchers have investigated the properties of base-activated materials. Zhang et al. [11] studied the high-temperature resistance of a slag ceramic powder base polymer added with oyster powder. Their research found that adding oyster shell powder to the sample can effectively reduce the high-temperature damage (800 °C) to the alkali-activated material.

The findings presented in this Special Issue contribute to the continuous improvement of the sustainability and durability of cement-based and alkali-activated materials. The cement industry holds enormous potential to contribute to global efforts to combat climate change. By working towards carbon peaking and carbon neutrality, we can create a world where cement-based and alkali-activated materials are part of the solution rather than creating carbon emissions in the future.
